# Aureolic Acid Group of Agents as Potential Antituberculosis Drugs

**DOI:** 10.3390/antibiotics9100715

**Published:** 2020-10-19

**Authors:** Julia Bespyatykh, Dmitry Bespiatykh, Maja Malakhova, Ksenia Klimina, Andrey Bespyatykh, Anna Varizhuk, Anna Tevyashova, Tatiana Nikolenko, Galina Pozmogova, Elena Ilina, Egor Shitikov

**Affiliations:** 1 Federal Research and Clinical Centre of Physical-Chemical Medicine, 119435 Moscow, Russia; d.bespiatykh@gmail.com (D.B.); maja_m@mail.ru (M.M.); ppp843@yandex.ru (K.K.); aliviense@gmail.com (A.V.); snow_grom@inbox.ru (T.N.); pozmge@gmail.com (G.P.); ilinaen@gmail.com (E.I.); egorshtkv@gmail.com (E.S.); 2 Institute of Fundamental Medicine and Biology, Kazan Federal University, 420008 Kazan, Russia; andyoctopus@mail.ru; 3 Gause Institute of New Antibiotics, 199021 Moscow, Russia; chulis@mail.ru; 4 Moscow Institute of Physics and Technology, Dolgoprudny, 141700 Moscow, Russia

**Keywords:** *Mycobacterium smegmatis*, TB treatment, Olivomycin, *Mycobacterium tuberculosis*, transcriptomic

## Abstract

*Mycobacterium tuberculosis* is one of the most dangerous pathogens. Bacterial resistance to antituberculosis drugs grows each year, but searching for new drugs is a long process. Testing for available drugs to find active against mycobacteria may be a good alternative. In this work, antibiotics of the aureolic acid group were tested on a model organism *Mycobacterium smegmatis*. We presumed that antibiotics of this group may be potential G4 ligands. However, this was not confirmed in our analyses. We determined the antimicrobial activity of these drugs and revealed morphological changes in the cell structure upon treatment. Transcriptomic analysis documented increased expression of *MSMEG_3743/soj* and *MSMEG_4228/ftsW*, involved in cell division. Therefore, drugs may affect cell division, possibly disrupting the function of the Z-ring and the formation of a septum. Additionally, a decrease in the transcription level of several indispensable genes, such as nitrate reductase subunits (*MSMEG_5137/narI* and *MSMEG_5139/narX*) and *MSMEG_3205/hisD* was shown. We concluded that the mechanism of action of aureolic acid and its related compounds may be similar to that bedaquiline and disturb the NAD+/NADH balance in the cell. All of this allowed us to conclude that aureolic acid derivatives can be considered as potential antituberculosis drugs.

## 1. Introduction

Tuberculosis (TB) caused by *Mycobacterium tuberculosis* is still an acute problem worldwide. The disease continues to take about a million lives every year [1]. The situation is complicated by the steady growth in a number of *M. tuberculosis* resistant strains. Multidrug and extensively drug-resistant tuberculosis pathogens are among them. An increasing number of strains resistant to all known antituberculosis drugs was witnessed in recent years [2,3]. Thus, the problem of development of new TB drugs is acute. Despite the fact that the numerous chemical libraries of synthetic and natural compounds have been exhaustively screened to identify new drugs, this process is arduous. Thus, only one new antituberculosis drug, bedaquiline, has been introduced recently [4,5].

The process of developing new drugs is a slow process. In this regard, the testing of available medications to search for active antimycobacterial drugs (drug repositioning) is important. The well-studied antitumor drugs (for example, antibiotics of the aureolic acid group) can be of particular interest. They bind to the GC-rich sites of the DNA minor groove and form complexes with Mg^2+^ [6]. In turn, their toxic effect primarily affects transcription and replication. If an antibiotic binds in the vicinity of a gene promoter, it prevents polymerase binding and subsequent transcription. Because *Mycobacterium* genomes are GC-rich, it can be assumed that antibiotics of the aureolic acid group may be active against mycobacteria [7].

GC enrichment of mycobacterial genomes also results in the appearances of G-quadruplexes (G4s), which are a spiral G-rich non-canonical form of DNA organization [8]. Indeed, G4s were found in the promoter regions of *M. tuberculosis*, and, consequently, G4 ligands (BRACO-19 and TMPyP4) inhibited growth of bacterial cells [9,10]. Thus, antibiotics of the aureolic acid group may also be potential G4 ligands.

In the presented study, we used the fast-growing and nonpathogenic *M. smegmatis* to investigate the effect of aureolic acid group antibiotics. G4 motifs in the genomes of *M. tuberculosis* and *M. smegmatis* were analyzed, and the ability of the aureolic acid group drugs to stabilize G4 motifs was tested. The effect of these antibiotics on mycobacterial cells was also determined on the transcriptomic level. We assume that Olivomycin A, a member of the aureolic acid group, can be considered as a potential antituberculous drug.

## 2. Results and Discussion

### 2.1. Inhibiting Effect of the Aureolic Acid Group Compounds

To evaluate the influence of aureolic acid derivatives on mycobacteria cells, the effects of Chromomycin A3 (CHR), Mithramycin A (MTR), and Olivomycin A were analyzed. TMPyP4 and BRACO-19 were used as controls. Previously, it was shown that the well-known G4 ligands, TMPyP4 and BRACO-19, bind and stabilize G4 motifs and inhibit growth of *M. tuberculosis*. Published data indicate the biological significance of genes that possess G-quadruplexes in this pathogen and also demonstrate that G4s are potential targets for the development of effective anti-TB drugs [9,10]. As such, members of the aureolic acid group can be considered as potential stabilizers for G4 motifs. 

Considering that *Mycobacterium tuberculosis* is rather a complex subject (in part, because of its slow growth), the effect of aureolic acid derivatives was tested on its close relative, *M. smegmatis* [11,12]. It has a similarly high genome GC content, but grows faster and is not pathogenic.

**Figure 1:**
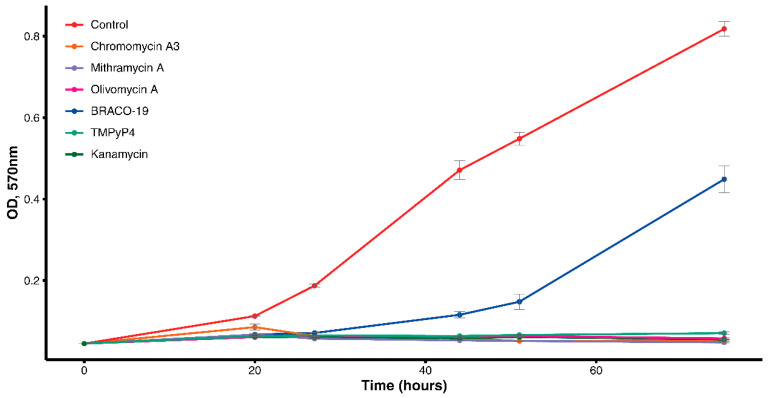
Growth curve of *M. smegmatis* with drugs and control culture.

To determine antimicrobial activity, the studied drugs were used in a concentration of 10 μM (corresponding to the previously used concentration of BRACO-19 for *M. tuberculosis* [9]). We documented that MTR, Olivomycin A, CHR, and TMPyP4 completely inhibited growth of *M. smegmatis* (Figure 1). In contrast, BRACO-19 significantly reduced growth of mycobacteria only in the first 27 h. However, by 75 h, the optical density was close to that of the control. Thus, aureolic acid drugs, along with TMPyP4, have antibacterial activity against *M. smegmatis*. Moreover, they appear to be more effective than previously described BRACO-19. 

**Figure 2:**
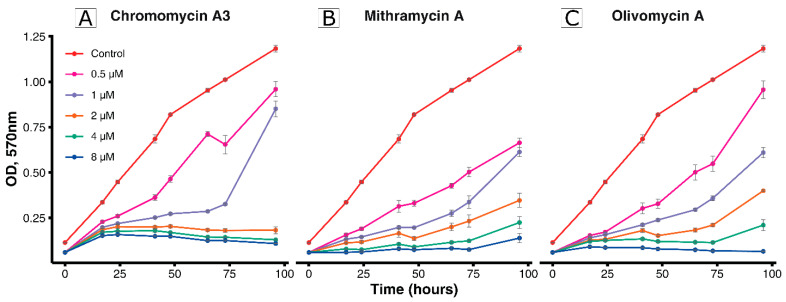
Evaluation of the drug’s inhibitory effect.

At the next step, the inhibitory effect for MTR, Olivomycin A, and CHR was evaluated (Figure 2). Concentrations of the drugs were chosen based on the values of sublethal doses (1–4 μM) previously reported [13,14]. Dose-dependent effect was observed for all drugs. It should be noted that, among antibiotics of the aureolic acid group, Olivomycin A is the least toxic drug and has the highest chemotherapeutic index [14]. In this way, in the further experiments for effects investigation Olivomycin A was used. 

**Figure 3:**
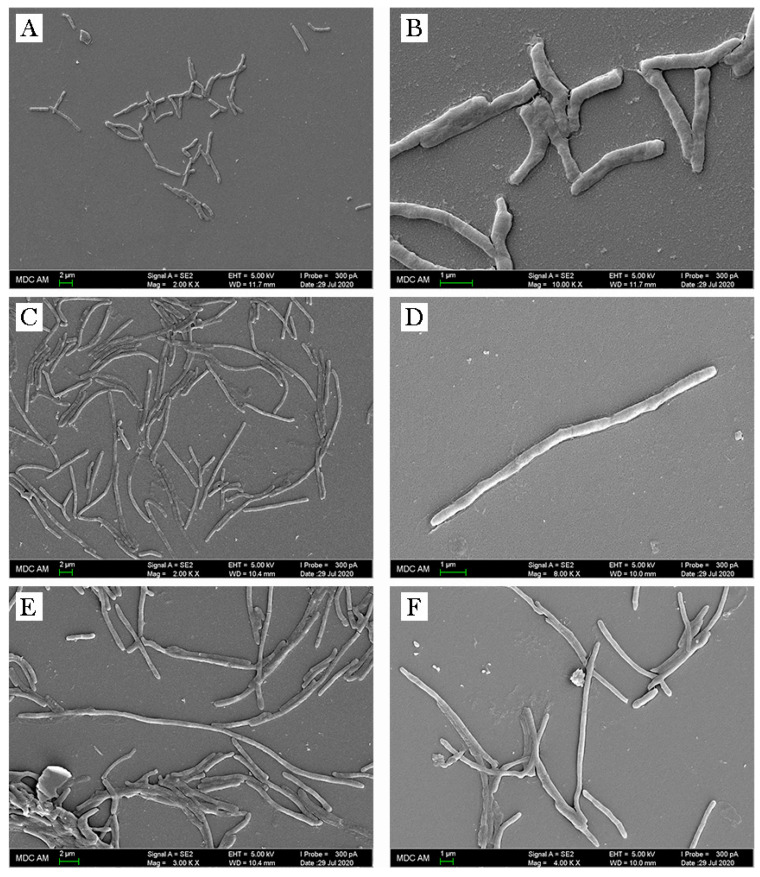
SEM images of *M. smegmatis*: (**A**,**B**) control cells; (**C**–**F**) cells growing with Olivomycin A.

The changes in morphology of Olivomycin-treated *M. smegmatis* cells were revealed by microscopy. Mycobacterial cells cultivated in the presence of sublethal doses of antibiotic (0.5 μM) were elongated and formed conglomerates not typical for control cells (Figure 3 and Supplementary Materials Figure S2). 

### 2.2. Identification of Putative G4 Motifs and Their Interaction with Aureolic Acid Derivatives

To detect and predict in vivo–folded G4s, a previously developed algorithm was used [15]. As a result, 834 and 703 G4s with the score over 40 were identified for *M. tuberculosis* and *M. smegmatis*, respectively (Supplementary Materials Table S1). As expected, the highest number of these motifs is located in the coding sequences. There were no statistically significant differences in the representation of motifs between organisms. 

Based on the results of the genomic study, we selected four high-scoring G4s from *M. smegmatis* for analysis of their interaction with aureolic acid derivatives (Table 1). G4 2s is localized in the promoter region of the *MSMEG_1900*, which encodes D-alanyl-D-alanine carboxypeptidase, involved in the peptidoglycan biosynthesis [16]. The product of 9s-harboring gene (*MSMEG_2124*) was an MIP-protein involved in the carbon transport [17]. Motifs 11s and 12s were attributed to the genes *MSMEG_2731* (DNA repair ATPase; presumably plays a role in transcription and translation [18]) and *MSMEG_2750* (iron-dependent repressor IdeR, iron concentration control [19]), respectively.

The secondary structures of all motifs were characterized by circular dichroism (CD) spectroscopy (Table 1). The spectrum of 12s contained major positive bands at 265 nm and pointed to parallel-stranded G4s with propeller loops and all guanines in the anti-conformation. In turn, remaining motifs carried characteristic features of both parallel and antiparallel G4s, suggesting a hybrid structure or a mixture (Supplementary Materials Figure S3).

To analyze the ligand-induced stabilization effect of aureolic acid derivatives, a FRET-melting assay was performed. Unfortunately, we did not find the stabilizing effect of the aureolic acid derivatives, while the known ligands have shown an effect (Table 1). The latter suggests that the aureolic acid derivatives are not G4-stabilizing ligands.

### 2.3. Transcriptomic Analysis and Correlation with CG Genome Composition

The transcriptomic analysis was carried out to determine the mechanism of Olivomycin A action and drug’s general influence on the cell. *M. smegmatis* cells cultivated in the presence of 0.5 μM of Olivomycin A were used for analysis. In total, 6612 and 6545 *M. smegmatis* transcripts were identified for experiment and control, respectively. Eight hundred and five genes were differentially expressed (at least a two-fold difference in their abundance), out of which Olivomycin A decreased transcription of 508 genes, and increased transcription of 297 genes (Supplementary Materials Table S2 and Figure S4).

These data were correlated with gene GC content, and no significant correlation was found for individual genes (Supplementary Materials Figure S5A), while such dependence was documented for the promoters of operons (R = 0.12, Supplementary Materials Figure S5B). We concluded that Olivomycin A binds to the promoter regions of the operons. 

For further analysis of differently expressed genes, the identified changes were assigned to non-specific and specific. Non-specific changes include the cell’s response to stress caused by the antibiotic [20,21]. For example, several studies have shown that antibiotics, such as β-lactams, quinolones, and aminoglycosides, can induce the production of reactive oxygen species (ROS) in bacteria [22,23]. At the same time, other changes that are observed only as a response to the described drugs or identified in this study for the first time are discussed further, as specific.

### 2.4. Non-Specific Changes of M. smegmatis in Response to Olivomycin A

According to the above assumption, non-specific changes included a reduced transcription of two-component systems and changes in the NAD+/NADH balance ratio (Table 2). Similar changes are observed in bacteria in response to different types of stress. Particularly, a reduced transcription of nitrate reductase subunits (*MSMEG_5137/narI* and *MSMEG_5139/narX*), involved in nitrogen metabolism, was detected. In addition, a significant decrease in transcription of *MSMEG_3205/hisD*, which is vital for mycobacteria, was revealed. HisD is a bifunctional enzyme that catalyzes the NAD+- and Zn2+-dependent conversion of l-histidinol (l-Hol) to l-histidine (l-His) through an l-histidinaldehyde (l-Hal) intermediate, with the concomitant reduction of 2 molecules of NAD+. The possibility of using *hisD* as a target for new antituberculosis drugs has also been reported previously [24]. As a result of such changes, reactive oxygen species (ROS) accumulate in the cell, since they are formed mainly through the transfer of electrons along the respiratory chain and the conversion of NADH into NAD+ [25]. In turn, changes in the NAD+/NADH ratio may imbalance intracellular redox potential [26]. This is also evidenced by the increased transcription of the genes encoding the ATP-binding transporters (*MSMEG_5008*, *MSMEG_6046*, *MSMEG_6052*, *MSMEG_1640*) and ATPases (*MSMEG_0615*, *MSMEG_5044*, *MSMEG_6058*). It was previously reported that ATP synthase operon had increased transcription in response to bedaquiline. It is safe to assume that molecular mechanisms, facilitating bactericidal effects of bedaquiline and Olivomycin, are similar, as both chemicals uncouple the respiration-driven ATP synthesis, leading to the collapse of the transmembrane pH gradient and dissipation of the proton-motive force [27].

Decreased transcription of several genes (*MSMEG_5392*/*kdpA*, *MSMEG_5393*/*kdpB*, *MSMEG_5394*/*kdpC*, *MSMEG_5395*/*kdpD*, and *MSMEG_5396*/*kdpE*) encoding the Kdp potassium transport system was detected in cell treated with Olivomycin. KdpE can bind to the promoter region of kdpFABC operon in *M. smegmatis* and regulate the osmotic pressure upshift, different intracellular ATP levels, and pH of the medium [28]. At the same time, KdpB is associated with KdpC that is essential for ATP hydrolysis [29]. In the presented study, the transcription of *MSMEG_5394/kdpC* was significantly reduced (8.5-fold).

We detected increased transcription of three genes (*MSMEG_2943*, *MSMEG_2944*, and *MSMEG_2945*), encoding the RuvABC protein complex. These proteins are involved in DNA repair. Differences in their transcription may also indicate the accumulation of ROS in the cell [30]. Additionally, increased transcription of *MSMEG_2740/lexA* was observed. It encodes a key enzyme of SOS response and DNA reparation [31]. We also detected a correlated increased transcription of the *MSMEG_2723/recA* gene associated with *lexA*. Similar changes have been observed in *M. tuberculosis* upon treatment with fluoroquinolones [32,33].

We further demonstrate a decreased transcription of genes encoding the cytochrome oxidase complex (*MSMEG_3231/cydD*, *MSMEG_3232/cydB*, and *MSMEG_3233/cydA*). The genes *cydA* and *cydB* encode two subunits of the cytochrome bd-oxidase, which belongs to the widespread prokaryote family of quinoloxidases. The *cydD* and *cydC* genes (located immediately after *cydB*) encode the ATP-binding transporters. Previously it was shown that deleting these genes in *M. smegmatis* does not cause cell death. At the same time, mutants show a significant decrease in metabolic fitness compared to the wild type [34]. Thus, in the present study, reduced transcription of these genes may also correlate with changes in bacterial growth (Figure 1).

### 2.5. Specific Changes of M. smegmatis in Response to Olivomycin A

Specific changes, induced by Olivomycin A, mainly concern defects in cell division (Table 2). In particular, we observed increased transcription of *MSMEG_3743/soj* gene participating in the formation of cell septum [35]. Previously, it was shown that hyperproduction of this protein leads to a disturbed cellular cycle and the formation of threadlike multinucleate cells [36], which is in agreement with our data (Figure 3). These changes may indicate that the drug is affecting cell division, possibly disrupting the function of the Z-ring and the formation of a septum.

Additionally, increased transcription of the *MSMEG_4228/ftsW* gene, which is involved in cell division and previously positioned as a potential target for anti-TB drugs [37], was shown. Increased transcription of a number of genes (*MSMEG_0438*, *MSMEG_0704*, *MSMEG_0806*, *MSMEG_5043*, *MSMEG_5879*, *MSMEG_6109*, and *MSMEG_6369*) encoding lipoproteins was also documented. For mycobacteria treated with antibiotics, which disrupt formation of the cell wall (such as Cycloserine, ethambutol, and isoniazid), elevated transcription of the transcriptional regulator *whiB2* was shown [38,39]. *Mycobacterium smegmatis* also overexpresses whiB2 during the transition to uncultivated forms [40]. In our study, we detected the increased expression of *MSMEG_1831/whiB2* upon Olivomycin treatment.

Transcription of *MSMEG_1941* gene encoding the helicase of the UvrD/Rep family was increased after the addition of Olivomycin in *M. smegmatis*. The UvrD1 binds Mg^2+^·ATP and the single-stranded DNA tail on which the helicase loads and translocates during duplex unwinding [41]. It was previously reported that *M. tuberculosis* UvrD1 and UvrD2 helicases are capable of resolving G4 motifs [42]. Thus, increased transcription of *MSMEG_1941* and also ATP-dependent helicase gene *MSMEG_1943* may signal a cellular attempt to resolve GC-rich regions bound to Olivomycin.

## 3. Materials and Methods 

### 3.1. Bacterial Strain, Growth Conditions, and Inhibition Assay

In this work, *Mycobacterium smegmatis* mc^2^ 155 strain was used. It was grown on 7H10 agar and in 7H9 broth both supplemented with 0.5% glycerol, 10% oleic acid albumin dextrose complex (Becton Dickinson, Franklin Lakes, NJ, USA). 

For growth inhibition assay, the culture was grown to mid-log phase (OD_570_ ~ 0.4) and diluted to obtain a bacterial count of ~5 × 10^5^ per mL for the assay. 

To antibacterial activity analysis, Olivomycin A, Mithramycin A, Chromomycin A3, BRACO-19, and TMPyP4 (all from Sigma-Aldrich, St. Louis, MO, USA) (Supplementary Materials Figure S1) were added to the cultures, at a final concentration of 10 μM. As a positive control, Kanamycin was used at a final concentration of 20 μM. Negative control samples were treated with the same volume of DMSO (a solvent for all chemicals above). The samples were cultivated in a flask (40 mL final volume) in 7H9 broth, with supplements, at 37 °C, with sharking (5 rpm) and 5% CO_2_. The optical density (OD) at 570 nm was measured during 75 h of incubation on Multiskan™ FC Microplate Photometer (Thermo Scientific™, Waltham, MA, USA). For inhibitory effect evaluation, Olivomycin A, MTR, and CHR were added to the cultures at a final concentration of 8, 4, 2, and 0.5 μM. OD_570_ were enumerated during 96 h of incubation in the same conditions.

For microscopy and transcriptomic analysis, *M. smegmatis* cells were cultivated with 0.5 μM Olivomycin A (experiment) or DMSO (control). The cultures (40 mL) were grown in three biological replicates, in cell culture flasks kept horizontally, at 37 °C, for 12 days, with constant shaking (5 rpm) and 5% CO_2_ until OD570 ~ 0.4. The bacterial suspension from each flask was split into 35 mL (for transcriptomic analysis) and 5 mL (for microscopy) aliquots, at room temperature (RT).

Samples for transcriptomic analysis were centrifuged at 3200× *g* for 10 min (RT) and cells pellets were frozen in liquid nitrogen and stored at −80 °C until further use. For microscopy analysis, cells were harvested by centrifugation, at 3500× *g*, at 4 °C, for 5 min, and washed twice with a phosphate buffer (pH 7.2). 

### 3.2. Microscopy

The washed cells were heat-fixed and stained, using the Ziehl–Neelsen method, as described previously [43]. Stained slides were visualized by using the Axio Observer microscope equipped with an AxioCam MTC digital camera system and Zen software (Carl Zeiss AG, Oberkochen, Germany). For scanning electron microscopy (SEM), fixed cells were processed as described elsewhere [44] and examined using a scanning electron microscopy multipurpose analytical complex Merlin (Carl Zeiss).

### 3.3. Transcriptomic Analysis

Total RNA was isolated from all *M. smegmatis* cultures, as previously described [45,46]. DNase treatment was carried out with TURBO DNA-free kit (Thermo Fisher Scientific, Waltham, MA, USA), in volumes of 100 µL, and further with the RNase-Free DNase Set (Qiagen, Hilden, Germany), according to the manufacturers’ protocol. RNA cleanup was performed with the RNeasy Mini Kit (Qiagen). The concentration and quality of the total RNA were checked by the Quant-it RiboGreen RNA assay (Thermo Fisher Scientific) and the RNA 6000 Pico chip (Agilent Technologies, Santa Clara, CA, USA), respectively.

Total RNA (1–2.5 µg) was used for library preparation as previously described [47]. Equimolar quantities of all libraries (12 pM) were sequenced by a high-throughput run on the Illumina HiSeq2500, using 2 × 100 bp paired-end reads and a 5% Phix spike-in control. In total, 104 million paired reads were obtained. The dataset of RNA-Seq analysis was deposited to the NCBI, with the project name PRJNA659121.

### 3.4. Bioinformatics Analysis of M. tuberculosis and M. smegmatis

Quality control of raw RNA-seq reads was carried out with FASTQC v0.11.5 [48]. Adapters and low-quality sequences were removed with the Trimmomatic v0.33 tool [49]. The Kallisto v0.46.0 [50] program was used to get reads pseudoalignments and abundance estimation. *Mycobacterium smegmatis* mc^2^ 155 complete genome (GenBank accession number: CP000480.1) was used as reference. Counts from Kallisto quantification output were extracted with the tximport v1.14.2 package [51]. Differential gene expression analysis was performed by using edgeR v3.26.8 [52] package, integrated in the Degust v4.1.1 [53] web-tool. Genes were considered significantly differentially expressed if they had false discovery rate cutoff (FDR) ≤ 0.05 and minimum expression fold change (FC) ≥ 2.

Putative G-quadruplexes (PGQs) in the genomes of *M. smegmatis* mc^2^ 155 (GenBank accession number: CP000480.1) and *M. tuberculosis* strain H37Rv (GenBank accession number: NC_000962.3) were predicted by G4-iM Grinder [15] with following search parameters: Complementary = TRUE, BulgeSize = 0, RunComposition = “G”, MaxRunSize = 4, MinRunSize = 3, MaxNRuns = 0, MinNRuns = 4, MaxIL = 0, MaxLoopSize = 15, MinLoopSize = 0. The score of 40 was used as a threshold.

Spearman correlation analysis was performed, to assess the relationship between GC content in intergenic regions and changes in gene-expression levels.

### 3.5. Circular Dichroism Spectroscopy and FRET-Melting Assay

Four G4-forming oligodeoxyribonucleotides (ODNs) labeled with 6-carboxyfluorescein (FAM) and Black Hole quencher 1 (BHQ) were purchased from Litekh (Moscow, Russia). The type of secondary structure in the working buffer (20 mM sodium-phosphate, pH 7.4, and 10 mM KCl) was verified by CD spectroscopy. The CD spectra were recorded, using a Chirascan spectrophotometer (Applied Photophysics, Letherhead, UK), equipped with a thermostated cuvette holder, at 15 °C. Prior to CD spectroscopy, each ODN sample (3 μM solution in the working buffer) was annealed rapidly, i.e., heated to 90 °C for 5 min and cooled on ice, to facilitate intramolecular folding. 

Melting curves for FRET-melting experiments were obtained by using a QuantStudio 5 PCR system (ThermoFisher Scientific) in the “step-hold” mode, with an average temperature ramp rate of 1.5 °C/min. Prior to the analysis, ODN samples (0.5 μM solutions in the working buffer) were annealed rapidly, and then the ligands were added to a final concentration of 10 μM. FAM fluorescence was registered every 0.3 °C, and the melting temperatures were determined from the first derivatives of the melting curves. 

## 4. Conclusions

Our data demonstrate antimycobacterial activity of Olivomycin A. We showed that it significantly inhibits the growth of *M. smegmatis*, the closest relative of *M. tuberculosis*. Transcriptomic analysis revealed a decrease in the transcription of several essential genes and an active cell response on the stress. The molecular mechanism of Olivomycin A activity may be similar to that of bedaquiline and go via distortion of the cellular NAD+/NADH balance. Additionally, (and similarly to ethambutol and isoniazid), the drug may cause cell-division defects.

Despite the drug’s affinity to GC, it is unable to stabilize the G4 motives. Thus, the action of the drug is most likely determined by its binding to the GC rich sites in the promoters of operons and inhibition of their transcription. Previously it was shown that Olivomycin is not toxic to humans and is currently used as an antitumor agent. All of this allowed us to conclude that Olivomycin A can be considered as a potential new antituberculosis drug in the future.

## Figures and Tables

**Table 1:** G4s from the genome of *M. smegmatis* and their ligand-induced changes.

Code	Sequence of the Labeled ODN, 5’-3’	Topology ^1^	Tm, °C ± 1	Delta Tm, °C ± 2				
				Olivomycin A	MTR	CHR	TmPyP4	BRACO19
**2sG**	FAM-GGGGAGGATCATGGGGCTCGGGGCGGGG-BHQ1	h-G4, p>a	48	0	0	0	37	22
**9sG**	FAM-GGGGCGGAGACAGGGGCGGGGTTGCCGGCGGGG-BHQ1	h-G4, p>a	54	0	0	0	43	37
**11sG**	FAM-GGGGAACGGGCCGGGGTGTTGGGTGGGGCGTGGGCCGGGGGGTGGGCTTGGGGG-BHQ1	h-G4, p>a	40	0	0	0	36	23
**12sG**	FAM-GGGGATGGGGTTGCCGAACGGGGAGGTGGTGGGG-BHQ1	p-G4	42	0	0	0	40	0

^1^ h-G4, hybrid G4; p-G4, parallel G4; ODN, oligodeoxyribonucleotides.

**Table 2:** Changes of *M. smegmatis* in response to Olivomycin A discussed in the text.

Locus_Tag	Gene	Homolog in *M. tuberculosis*	Product	Functional Category	COG Classification	KEGG Classification	FC (Exp/Control)
**Non-Specific Changes**							
MSMEG_0615	eccA3	Rv0282	ATPase AAA	cell wall and cell processes	-	-	5.48155
MSMEG_1640	-	Rv3362c	ATP/GTP-binding protein	conserved hypotheticals	General function prediction only	Not assigned to any KEGG category	2.32442
MSMEG_2723	recA	Rv2737c	recombinase A	information pathways	Replication, recombination and repair	Not assigned to any KEGG category	2.11516
MSMEG_2740	lexA	Rv2720	LexA repressor	regulatory proteins	Signal transduction mechanisms | Transcription	Not assigned to any KEGG category	2.22158
MSMEG_2943	ruvC	Rv2594c	Holliday junction resolvase	information pathways	Replication, recombination and repair	Replication and repair	2.02021
MSMEG_2944	ruvA	Rv2593c	Holliday junction DNA helicase RuvA	information pathways	Replication, recombination and repair	Replication and repair	2.2836
MSMEG_2945	ruvB	Rv2592c	Holliday junction DNA helicase RuvB	information pathways	Replication, recombination and repair	Replication and repair	2.09412
MSMEG_3205	hisD	Rv1599	histidinol dehydrogenase	intermediary metabolism and respiration	Amino acid transport and metabolism	Amino acid metabolism	−2.10763
MSMEG_3231	cydD	Rv1621c	cysteine ABC transporter permease/ATP-binding protein	intermediary metabolism and respiration	Energy production and conversion | Post-translational modification, protein turnover, and chaperones	Membrane transport	−2.5954
MSMEG_3232	cydB	Rv1622c	cytochrome D ubiquinol oxidase subunit II	intermediary metabolism and respiration	Energy production and conversion	Energy metabolism| Signal transduction	−2.53386
MSMEG_3233	cydA	Rv1623c	cytochrome D ubiquinol oxidase subunit1	intermediary metabolism and respiration	-	-	−2.1669
MSMEG_5008	-	Rv1273c	ABC transporter ATP-binding protein	cell wall and cell processes	Defense mechanisms	Not assigned to any KEGG category	2.04337
MSMEG_5044	-	Rv1251c	ATPase	conserved hypotheticals	General function prediction only	Not assigned to any KEGG category	2.1844
MSMEG_5137	narI	-	respiratory nitrate reductase subunit gamma	-	-	Nitrogen metabolism	−2.28108
MSMEG_5139	narH	Rv1162	nitrate reductase subunit beta	intermediary metabolism and respiration	-	-	−2.0609
MSMEG_5392	kdpA	Rv1029	potassium-transporting ATPase A	cell wall and cell processes	Inorganic ion transport and metabolism	Signal transduction	−3.84888
MSMEG_5393	kdpB	Rv1030	potassium-transporting ATPase subunitB	cell wall and cell processes	Inorganic ion transport and metabolism	Signal transduction	−3.62873
MSMEG_5394	kdpC	Rv1031	potassium-transporting ATPase subunitC	cell wall and cell processes	Inorganic ion transport and metabolism	Signal transduction	−8.51123
MSMEG_5395	kdpD	Rv1028c	sensor protein KdpD	regulatory proteins	Signal transduction mechanisms	Not assigned to any KEGG category	−2.399
MSMEG_5396	kdpE	Rv1027c	KDP operon transcriptional regulatory protein KdpE	regulatory proteins	Signal transduction mechanisms | Transcription	Signal transduction	−2.5025
MSMEG_6046	-	-	ABC transporter ATP-binding protein	-	Not in COGs	Not assigned to any KEGG category	2.0133
MSMEG_6052	-	-	ABC transporter ATP-binding protein	-	Not in COGs	Not assigned to any KEGG category	2.39154
MSMEG_6058	-	-	cadmium transporting P-type ATPase	-	Not in COGs	Not assigned to any KEGG category	2.8424
**Specific Changes**							
MSMEG_0438	-	Rv0265c	periplasmic binding protein	cell wall and cell processes	Inorganic ion transport and metabolism	Membrane transport	2.68116
MSMEG_0704	lpqJ	Rv0344c	LpqJ protein	cell wall and cell processes	Not in COGs	Not assigned to any KEGG category	2.27771
MSMEG_0806	lpqL	Rv0418	hydrolase	cell wall and cell processes	General function prediction only	Not assigned to any KEGG category	2.24391
MSMEG_1831	whiB2	Rv3260c	transcription factor WhiB	regulatory proteins	Not in COGs	Not assigned to any KEGG category	2.27169
MSMEG_1941	-	-	helicase, UvrD/Rep family protein	-	Not in COGs	Not assigned to any KEGG category	2.21769
MSMEG_1943	-	Rv3201c	ATP-dependent DNA helicase	information pathways	Replication, recombination and repair	Replication and repair	2.21528
MSMEG_3743	soj	Rv1708	SpoOJ regulator protein	cell wall and cell processes	Cell cycle control, cell division, chromosome partitioning	Not assigned to any KEGG category	2.05016
MSMEG_4228	ftsW	Rv2154c	cell division protein FtsW	cell wall and cell processes	Cell cycle control, cell division, chromosome partitioning	Not assigned to any KEGG category	2.3923
MSMEG_5043	lprE	Rv1252c	LprE protein	cell wall and cell processes	Not in COGs	Not assigned to any KEGG category	2.35097
MSMEG_5879	lpqR	Rv0838	D-alanyl-D-alanine dipeptidase	cell wall and cell processes	Cell wall/membrane/ envelope biogenesis	Signal transduction| Drug resistance	2.30757
MSMEG_6109	lpqG	Rv3623	LpqG protein	cell wall and cell processes	Function unknown	Not assigned to any KEGG category	2.19376
MSMEG_6369	rfbD	Rv3783	O-antigen export system, permease	cell wall and cell processes	Carbohydrate transport and metabolism | Cell wall/membrane/ envelope biogenesis	Not assigned to any KEGG category	2.11427

## Supplementary Materials

The following are available online at https://www.mdpi.com/2079-6382/9/10/715/s1, Figure S1: Chemical structures of aureolic acid group antibiotics. (A) Chromomycin A3 (CHR), (B) Mithramycin A (MTR), (C) Olivomycin A, (D) BRACO-19, and (E) TMPyP4. Figure S2: Light microscope photographs of *M. smegmatis* cells stained using the Ziehl–Neelsen method. (A) control cells and (B) cells growing with Olivomycin A. Scale bar = 5 μm. Figure S3: Circular dichroism spectra of G4s from *M. smegmatis.* Figure S4: Volcanoplot of differential expression genes. Figure S5: Correlation between gene GC content and (A) individual genes, and (B) operon’s promoters. Table S1: Characterization of G4s sequences in the Mycobacteria genomes. Table S2: List of identified and quantified transcripts for *M. smegmatis*.

## Abbreviations

CHR	Chromomycin A3
MTR	Mithramycin A
TB	Tuberculosis
G4	G-quadruplexes
CD	Circular dichroism
PGQ	Putative G-quadruplexe
ODN	Oligodeoxyribonucleotide
OD	Optical density
FDR	False discovery rate
FC	Fold change
SEM	Scanning electron microscopy
