# Supplementary figures and images for: Aureolic Acid Group of Agents as Potential Antituberculosis Drugs
Source: Antibiotics (Basel). 2020 Oct 19;9(10):715. doi: 10.3390/antibiotics9100715 (PMC7650759; doi:10.3390/antibiotics9100715)

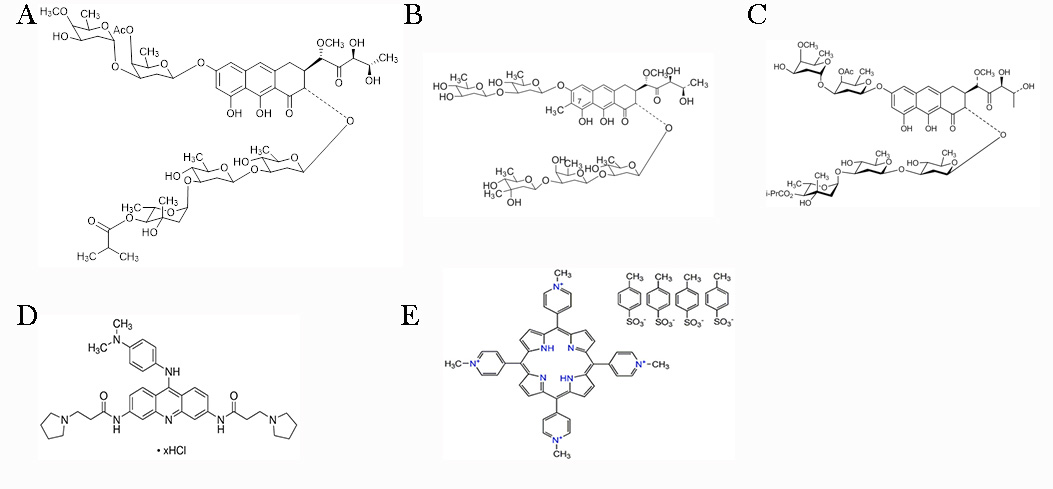

Supplement: Supplementary file 1 [file antibiotics-09-00715-s001.zip › Figure S1.jpg]

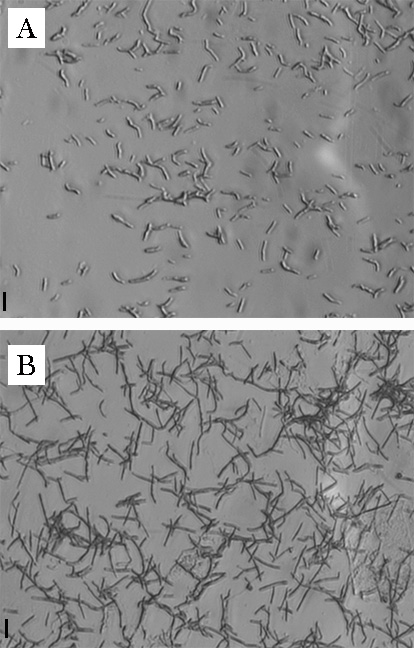

Supplement: Supplementary file 1 [file antibiotics-09-00715-s001.zip › Figure S2.jpg]

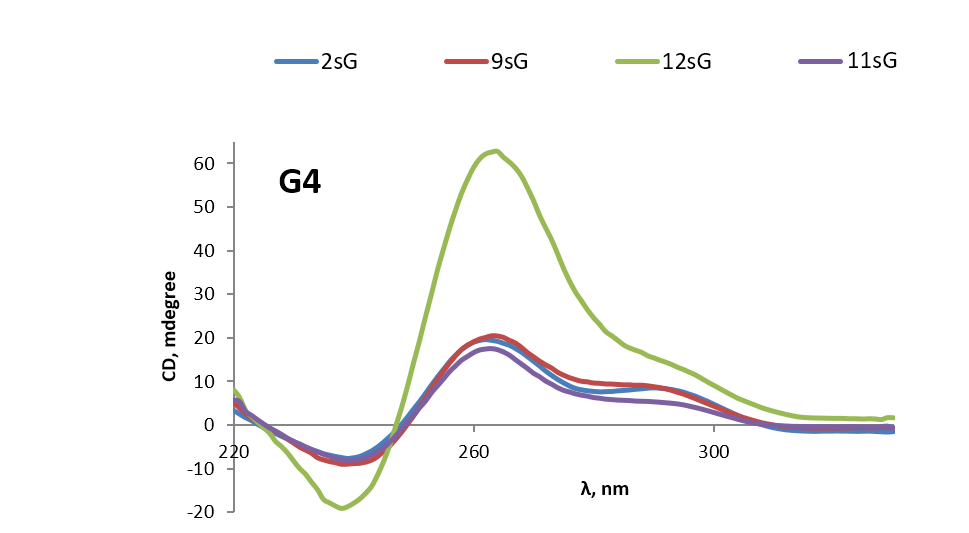

Supplement: Supplementary file 1 [file antibiotics-09-00715-s001.zip › Figure S3.png]

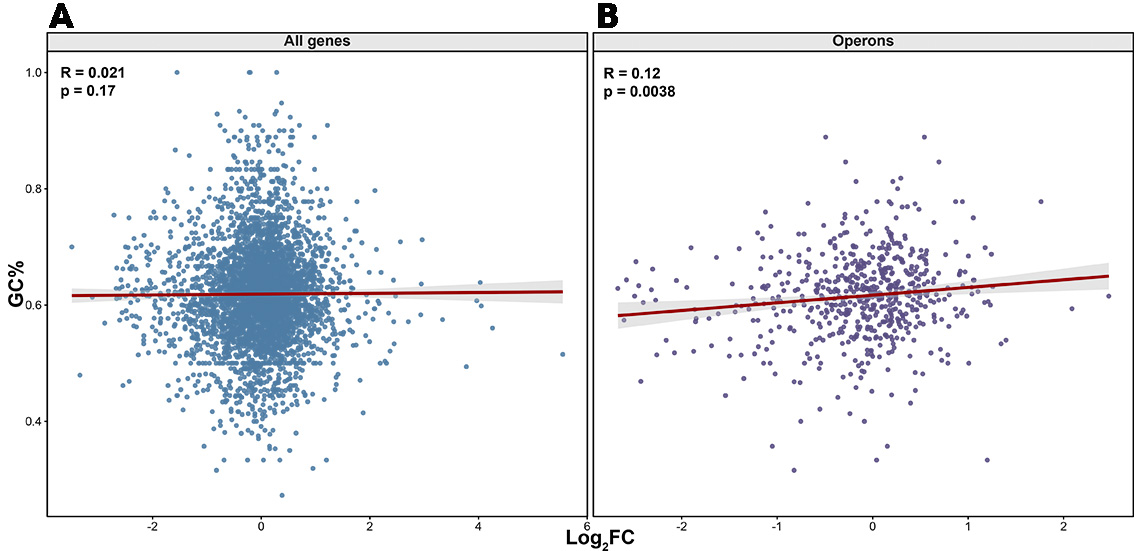

Supplement: Supplementary file 1 [file antibiotics-09-00715-s001.zip › Figure S5.jpg]
